# Review of Epidural Spinal Cord Stimulation for Augmenting Cough after Spinal Cord Injury

**DOI:** 10.3389/fnhum.2017.00144

**Published:** 2017-03-28

**Authors:** Jan T. Hachmann, Jonathan S. Calvert, Peter J. Grahn, Dina I. Drubach, Kendall H. Lee, Igor A. Lavrov

**Affiliations:** ^1^Department of Neurologic Surgery, Mayo ClinicRochester, MN, USA; ^2^Mayo Clinic Graduate School of Biomedical SciencesRochester, MN, USA; ^3^Department of Physiology and Biomedical Engineering, Mayo ClinicRochester, MN, USA; ^4^Department of Physical Medicine and Rehabilitation, Mayo ClinicRochester, MN, USA

**Keywords:** spinal cord injury (SCI), functional electrical stimulation (FES), spinal cord stimulation, epidural spinal cord stimulation, neuromodulation, neuroprosthetics, neurorestoration, cough restoration

## Abstract

Spinal cord injury (SCI) remains a debilitating condition for which there is no cure. In addition to loss of somatic sensorimotor functions, SCI is also commonly associated with impairment of autonomic function. Importantly, cough dysfunction due to paralysis of expiratory muscles in combination with respiratory insufficiency can render affected individuals vulnerable to respiratory morbidity. Failure to clear sputum can aggravate both risk for and severity of respiratory infections, accounting for frequent hospitalizations and even mortality. Recently, epidural stimulation of the lower thoracic spinal cord has been investigated as novel means for restoring cough by evoking expiratory muscle contraction to generate large positive airway pressures and expulsive air flow. This review article discusses available preclinical and clinical evidence, current challenges and clinical potential of lower thoracic spinal cord stimulation (SCS) for restoring cough in individuals with SCI.

## Cough Insufficiency in Spinal Cord Injury—Epidemiology and Clinical Significance

Spinal cord injury (SCI) is a complex condition resulting in numerous neurologic deficits in the sensorimotor and autonomic systems that can have a profound impact on functional performance, quality of life and independence. Among these potentially debilitating disorders are respiratory compromise and cough dysfunction. Inspiratory insufficiency, common with SCI of the cervical or thoracic spinal cord, is caused by respiratory muscle weakness, paralysis of chest wall muscles, decreased chest wall compliance, diminished truncal muscle tone and altered posture. Inspiratory dysfunction can restrict ventilation, precipitate atelectasis and contribute to respiratory complications. Similarly, paralysis of the expiratory muscles can impair or abolish the ability to cough. A recent multi-center survey of 147 subjects found that 30.9% of individuals with SCI had significant impairment of forced vital capacity (FVC) and over one-third (35.9%) reported poor to moderate cough strength (Postma et al., 2016). In a similar survey, 68% of 180 individuals with SCI reported respiratory symptoms, including breathlessness, increased prevalence of ineffective cough, wheezing and awareness of phlegm (Spungen et al., 1997).

Impaired respiratory integrity and dysfunctional cough not only impair respiratory performance and sense of well-being, but are also important contributors to respiratory morbidity and mortality in this population. Cough dysfunction disrupts the physiological clearance of bronchoalveolar secretions and phlegm, which is critical for pulmonary homeostasis. Sympathetic dysfunction and resulting unopposed secretions and phlegm, which is critical for pulmonary homeostasis. Sympathetic dysfunction and resulting unopposed parasympathetic innervation increases respiratory secretions, as well as contributes to bronchoconstriction (Schilero et al., 2005; Burns, 2007). Retained secretions congest the bronchial system, increase airway resistance, impede alveolar gas transport, and increase vulnerability to pathogens (Kowalski et al., 2007; Lim et al., 2007; Jefferson et al., 2010). Consequently, to mobilize secretions, individuals with SCI generally rely on various manual or machine-assisted techniques, including “quad coughing” (i.e., manually-assisted cough), percussion, mechanical insufflation-exsufflation, high-frequency chest wall oscillation, or suctioning (Burns, 2007). Despite these techniques, affected individuals remain at increased risk for both incidence and severity of pulmonary complications, as well as increased risk of fatality. Thus, even minor upper respiratory tract infections, such as uncomplicated bronchitis, can predispose people with SCI to rapid exacerbation and secondary pneumonia (Burns, 2007), which remains a common cause of death in the SCI population with a 37-fold increased standardized mortality ratio of compared to the general population (and up 150-fold for tetraplegia; DeVivo et al., 1999). Respiratory complications are a leading cause of hospitalization for individuals with SCI after genitourinary disorders and skin conditions (Cardenas et al., 2004) and restoration of cough presents a critical challenge for SCI patients. Importantly, none of the currently clinically-available neuroprosthetic devices for respiratory restoration are designed for restoring expiratory muscle function for cough (Ragnarsson, 2008; Collinger et al., 2013). Successful development of a device for functional electrical stimulation (FES)-evoked cough depends first on understanding the neuromuscular mechanisms involved in generating physiological cough.

## Neurophysiology of Forced Expiration and Cough

Natural exhalation of basal tidal volumes during resting respiration occurs passively through the inherent tension recoil force of the lungs and chest following active diaphragmatic contraction and thoracic expansion during inspiratory phase. Subsequent relaxation and passive compression of the intrapulmonary gas volumes slowly reverses the pressure gradient between the lungs and external atmospheric pressure generating gradual airflow out of the lungs. However, during states of elevated respiratory demand (e.g., hypoxia, exertion and increased airway resistance) expiration requires active muscle contraction to augment expiratory performance and maintain adequate air expulsion so as to sustain higher respiratory rates and volumes. Forced expiration requires contraction of various synergistic expiratory muscles (e.g., abdominal, pectoral and internal intercostal muscles). The abdominal muscles compress the abdominal cavity, increasing intraabdominal pressure and forcing the diaphragm upwards. The accessory expiratory muscles, most notably the internal intercostal muscles, force the ribs downward causing the thoracic wall to descend. These opposing movements compress the intrathoracic space and thus augment expiratory airway pressure and effective air flow rate.

**Figure 1:**
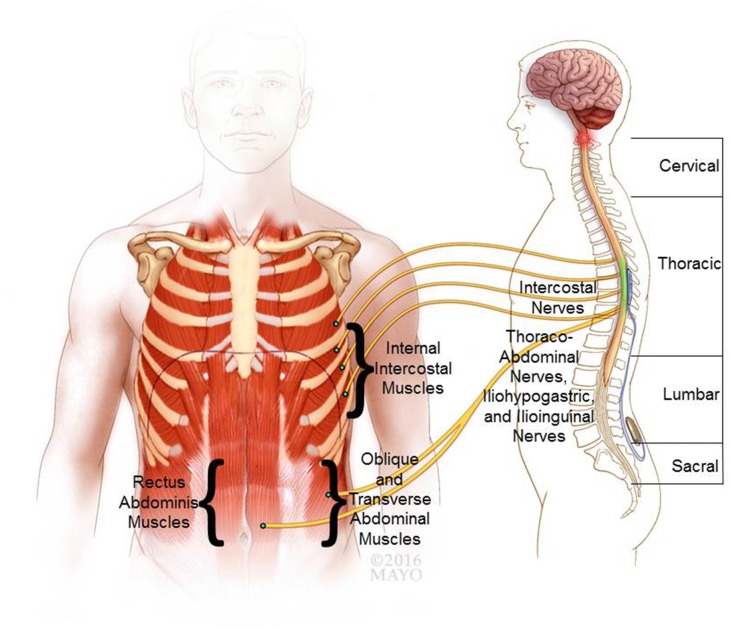
**Neurophysiological basis of cough and epidural stimulation of the lower thoracic spinal cord.** The expiratory internal intercostal muscles are innervated by their respective segmental intercostal nerves and the abdominal muscles are innervated via the lower intercostal branches (thoracoabdominal nerves), as well as regional branches from the lumbar plexus (i.e., ilioinguinal and iliohypogastric nerves). Based on the reviewed literature a putative target location for an epidural electrode is shown for lower thoracic spinal cord stimulation (SCS). Co-stimulation of the lower thoracic (e.g., T9) and upper lumbar (e.g., L1) spinal levels have shown to recruit the expiratory muscles and generate high peak expiratory pressures and air flow.

Physiologically, cough presents an airway-defensive mechanism characterized as a three-phase motor act that includes: (1) “inspiratory phase” with large-volume inspiration; (2) “compressive phase” with rapid, forceful and coordinated expiratory muscle contraction for thoracic compression against a closed glottis; and (3) “expulsive phase” with opening of the glottis for expulsive air movement. Thereby, high peak expiratory pressure and flow rate (i.e., velocity) are generated to facilitate effective airway clearance (Loudon and Shaw, 1967). Maximal volitional efforts of healthy individuals can typically generate positive airway pressures (Paw) in the range of ~200 cm H_2_O for males and ~150 cm H_2_O for females (Leith et al., 1986). Cough performance is significantly impaired after cervical SCI (DiMarco et al., 2009a,b). Lower thoracic spinal cord stimulation (SCS) aims to restore the compressive and expulsive phases of cough and therefore still relies on preceding synergistic volitional inspiratory effort for maximal efficacy (Loudon and Shaw, 1967). In healthy individuals cough can be either volitionally generated or triggered reflexively in response to inhalation of irritating stimuli via an afferent reflex pathway that is mediated via neuronal networks in the brainstem projecting to the expiratory muscles, as well as inspiratory and laryngeal muscles (Loudon and Shaw, 1967; Iscoe, 1998). These descending higher-order control axons branch extensively ipsilaterally and contralaterally throughout the thoracic (T1–T12) and upper lumbar (L1–L3) spinal cord (Monteau and Hilaire, 1991). This intraspinal network coordinates the complex muscular activation patterns required for cough generation (Merrill, 1974; Figure 1). The internal intercostal muscles are innervated by intercostal nerves arising segmentally from their respective thoracic nerve roots. Similarly, the upper abdominal muscles are mainly innervated segmentally via the lower intercostal nerves, whereas the lower abdominal muscles are innervated via regional branches originating in the lumbar plexus (i.e., ilioinguinal and iliohypogastric nerves). Importantly, the upper internal intercostal muscles (i.e., T1–T6 segments) are relatively thin (Monteau and Hilaire, 1991) and provide only marginal biomechanical contribution to the production of airway pressure (Budzinska et al., 1989). In contrast, the lower internal intercostal muscles constitute a much larger cross-sectional area and are thus responsible for the majority of active chest wall depression (DiMarco et al., 1993). Finally, the pectoralis major muscles, which contribute to compression of the upper thoracic cavity, receive regional innervation from the brachial plexus (i.e., C5–T1 spinal levels) via medial and lateral pectoral nerves. The key effector neural control structures that innervate the major muscular contributors to cough are thus located primarily in the lower thoracic and upper lumbar spinal segments (Monteau and Hilaire, 1991). However, in cases of cervical or thoracic SCI, the primary bulbospinal input from the primary cough centers in the brain stem to the downstream effector lower motor neurons (LMNs) is disrupted resulting in expiratory muscle paralysis (Davis and Plum, 1972), which diminishes or abolishes the cough performance. Moreover, paralysis of the abdominal muscles can result in unfavorable paradoxical outward movement of the abdominal wall during the compressive and expulsive phases of cough that can hinder and diminish effective pressure generation (Estenne and Gorini, 1992). Additionally, uncoordinated isolated apical muscle contraction (i.e., segments with retained innervation proximal to the lesion) without synergistic co-contraction of basal muscular components can restrict thoracic pressure spikes to the apical segments resulting in limited dynamic airway compression. In turn, this may impede effective airflow patterns and impair effective clearance of more peripheral airways (Estenne et al., 1994). To ameliorate some of these mechanical restrictions associated with expiratory paralysis, a combination of specific muscle training and external mechanical abdominal binding has shown mild improvement in cough effectiveness in tetraplegia (Estenne and De Troyer, 1990; Van Houtte et al., 2006). However, efficacy and functional gains of manual, mechanical, or device-assisted approaches remain far from optimal with only modest improvements in expiratory cough performance. Therefore, more efficacious solutions are needed to restore effective cough for SCI population (Estenne and Gorini, 1992; Estenne et al., 1994).

## Restoring Cough Through Functional Electrical Stimulation (FES)

Augmentation and restoration of cough presents a challenge for available neuroprosthetic approaches. Anatomically remote muscles and disseminated innervation make the involved neuromuscular components poorly accessible via conventional peripheral FES-approaches. Nonetheless, there have been efforts to provide some augmentation via relatively basic peripheral stimulation. Transcutaneous stimulation of the abdominal muscles has demonstrated moderate improvement in maximal expiratory pressure (MEP), peak expiratory flow (PEF) and FVC. This approach has also reduced secondary pulmonary complications in individuals with tetraplegia (Linder, 1993; Cheng et al., 2006; Lee et al., 2008). Paired surface electrodes targeting the posterolateral portion of the trunk have been found to provide improved benefit (Lim et al., 2007). For example, Butler et al. (2011) found that surface stimulation of the posterolateral abdominal muscles can achieve two- to three-fold higher expiratory performance over previous targeting approaches, improving peak and mean expiratory flow by 36% and 80%, respectively, as well as augmenting tidal volumes by 41%. Supplementing abdominal surface stimulation with a mechanical abdominal binder provided some additive benefit, albeit generating only an additional 15%–18% PEF (Lin et al., 1998). Finally, Gollee et al. (2007) developed a control system for synchronizing abdominal stimulation with the subject’s volitional breathing activity. Using this automated trigger system, the authors reported successful augmentation of spontaneous respiration and cough with improvements in tidal volumes by 9%–71% and in cough peak flow (CPF) by 31%–54% (Gollee et al., 2007). However, while these and other studies (Taylor et al., 2002; Spivak et al., 2007; Hascakova-Bartova et al., 2008) have shown some degree of improvement in cough performance, restoring performance to levels of healthy individuals without SCI utilizing available techniques has yet to be achieved.

Therefore, it is paramount that new approaches to safe and effective neuroprosthetic devices for a more comprehensive restoration of cough and respiratory function be investigated. Important to consider in this effort is the fact that central stimulation may be able to recruit relevant effector thoracolumbar spinal networks that although cut off from brainstem cough control centers, nonetheless remain excitable below the level of the lesion. These retained circuits could potentially be leveraged for coordinating downstream segmental motor control. Over the past decades, epidural stimulation of the upper thoracic spinal cord has shown promise for augmenting inspiratory performance via (external) intercostal pacing (Decima et al., 1967, 1969; Decima and von Euler, 1969; DiMarco and Kowalski, 2009, 2010; DiMarco et al., 2009b). Similarly, epidural stimulation of the lower thoracic spinal cord has emerged as a promising new approach for evoking comprehensive and forceful recruitment of the expiratory muscles (DiMarco et al., 1999a,b, 2006, 2009a,b; DiMarco and Kowalski, 2008). Given these findings, it may be beneficial to consider incorporating central stimulation into the development of neural prostheses for cough enhancement that are capable of recruiting primary and accessory expiratory muscles to facilitate effective pulmonary clearance and improve respiratory health.

## Spinal Cord Stimulation of the Lower Thoracic Spinal Cord for Cough Restoration

### Preclinical Experience

Interest in epidural stimulation for cough restoration emerged in the late 1990s based on pioneering research by DiMarco et al. (1995, 1999b) who showed in canine animal models that epidural stimulation of the thoracolumbar spinal cord could evoke robust recruitment of expiratory muscles. Systematic stimulation along the rostrocaudal extent of the thoracolumbar spinal cord (canines: 13 thoracic segments and 7 lumbar segments) revealed that stimulation at the T9-level generated the largest changes in positive airway pressure (Paw) and PEF rate, the two primary performance parameters for measuring cough efficacy. Stimulation at the T9-level evoked near-optimal recruitment of the lower intercostal muscles (7th–11th interspaces), as well as upper portions of the abdominal muscles (external oblique, rectus abdominis and transversus abdominis muscles; DiMarco et al., 1999a,b). Interestingly, stimulation evoked cough diminished when moving in both the cranial and caudal directions along the neural axis away from the T9-level. Stimulation of more cephalad targets (e.g., T7-level) evoked predominant activation of the parasternal intercostals and 5th and 6th intercostal muscles, which make a negligible contribution to expiratory performance (Budzinska et al., 1989; Monteau and Hilaire, 1991). Stimulation of more caudal spinal levels (e.g., T11-level and below) resulted in relatively isolated recruitment of the associated segmental abdominal portions without co-activating intercostal muscles. Relative to the maximal Paw of 57 ± 3 cm H_2_O evoked at the T9-level, evocable expiratory pressures (Paw) progressively declined in caudal direction to 75%, 39% and 21% of maximal Paw at the T11-, T13-, and L2-levels, respectively. Even stimulation at the T9-level resulted in incomplete expiratory recruitment because it did not activate the more caudal portions of the abdominal muscles at clinically feasible stimulus amplitudes (DiMarco et al., 1999b). However, co-stimulation of the T13- and L1-segments was able to recruit the middle and lower portions of the abdominal muscles.

Thus, dual contact-stimulation did achieve a more comprehensive recruitment of these differentially innervated muscular components. Stimulation of the T9-level could effectively activate the upper portion of the abdominal muscles and the biomechanically relevant lower intercostal muscles, while stimulation at the T13-level provided complementary recruitment of the middle and lower abdominals. In this way, dual-contact stimulation was capable of markedly augmenting maximal expiratory performance by an additional ~30% elevating maximal Paw to 80 ± 3 cm H_2_O (compared to 57 ± 3 cm H_2_O at T9 and 22 ± 3 cm H_2_O at T13, respectively; DiMarco et al., 1995, 1999a,b, 2002).

Subsequent quantitative analysis showed that the oblique muscles accounted for the majority of expiratory performance at the T9-level with approximately 50% of total evoked Paw, followed by the transversus abdominis with 20%, the internal intercostals with 15%, and finally, the rectus abdominis with <7%. These results were somewhat inconsistent with prior evidence from both animal models and human studies that held that it was the transverse rather than oblique abdominal muscles that made the greatest contribution to expiratory pressure generation (Gilmartin et al., 1987; De Troyer et al., 1989, 1990). The authors concluded that the observed variation in relative contributions could likely be attributed to underlying differences in recruitment patterns between physiological activation and stimulation-evoked expiratory function (DiMarco et al., 1999a). Interestingly, dual-contact stimulation at the T9- and T13-levels shifted the contribution ratio moderately in favor of the transversus abdominis muscles. While the contribution of the oblique muscles remained relatively stable, accounting for 45% of total Paw, the relative contribution of the transverse abdominals increased to 42% of total Paw. Although there were no major adverse effects, there was some evidence of undesired spillover stimulation causing nonspecific co-activation of the inspiratory intercostals, albeit with negligible opposing force (<3 cm H_2_O; DiMarco et al., 1999a).

Recently, a chronic mini-pig model of SCI was used to evaluate the long-term effects of lower-thoracic SCS via epidural platinum/iridium wire electrodes for evoking cough restoration. The study found that bipolar charge-balanced stimulation across the lower thoracic contacts (i.e., T9- and T12-spinal levels) evoked near-maximal cough performance at stimulus parameters of 40 V and 50 Hz with 0.2 ms pulse duration. At 3 months post implantation, there was no evidence of electrode corrosion and only minimal signs of tissue reaction (e.g., fibrous electrode encapsulation, minimal inflammatory cells). However, stimulation at such high amplitude also resulted in spillover current spread, which evoked adverse motor effects (i.e., contraction of hind limb muscles; Kowalski et al., 2016a).

### Effects of Denervation and Central Stimulation on Muscle Fiber Plasticity

It is well-known that upon denervation, muscles, including those for respiration and cough, undergo rapid disuse atrophy resulting in reduction of muscle weight and cross-sectional area and consequent diminished strength and endurance (Lieber et al., 1986; Jiang et al., 1990; Stein et al., 1992; Bickel et al., 2004). Muscle fibers not only atrophy, they can also undergo plasticity changes, such as transformation of fiber type, generally towards a uniform and fatigable type-II fiber microstructure (i.e., “atrophy type”). However, stimulation can trigger tissue remodeling and stimulation-evoked adaptation plasticity effects, typically transforming fiber microstructure into either predominantly type-I- or type-II- fiber type expression depending on the stimulus parameters applied. For example DiMarco and Kowalski (2008) have shown in a feline animal model of SCI that even short intermittent bouts of lower thoracic (T10-level) SCS at 50 Hz twice daily for 15 min could successfully prevent atrophic changes and disadvantageous fiber type remodeling. Cough performance was stable over periods of 6 months with persistently high stimulation-evoked airway pressures. There were no significant changes in muscle weight or fiber type microstructure in the external oblique, internal oblique, transverse abdominal, internal intercostal, or rectus abdominis muscles, indicating that mixed fiber-type structure was maintained. It thus appears that stimulation at intermediate frequencies (e.g., 50 Hz) may help maintain the cross-sectional area and force-generating capacity of the targeted muscles while preserving the dynamic fiber type histostructure required for optimal balance of contraction force and endurance.

### Clinical Translation and Long-Term Efficacy

Based on these promising preclinical findings and on general clinical experience with FDA-approved commercially-available devices for epidural stimulation, this new paradigm was translated relatively quickly and successfully into the clinical investigative setting (DiMarco et al., 1999a,b, 2006, 2009a,b; DiMarco and Kowalski, 2008). A successful neuroprosthetic approach that was virtually identical to that used in the canine model demonstrated that the spinal cord anatomy and linear distance between thoracolumbar segments of the canine model were reasonably comparable to that of the human (DiMarco et al., 1999b). In the initial proof-of-concept study in 2006, a 52-year-old man with incomplete tetraplegia (American Spinal Injury Association [ASIA] Impairment Scale-C) and significant caregiver-dependent cough insufficiency was successfully treated with lower thoracic SCS following the established preclinical canine paradigm. Epidural stimulating electrodes were implanted via partial hemilaminectomies and introduced into the posterior epidural space under fluoroscopic guidance to target the T9, T11, and L1 spinal segments. Additionally, a stimulator/RF-receiver was implanted in a subcutaneous pocket over the anterior chest wall. Consistent with the overall clinical experience from electrophrenic respiration, the subject first had to undergo muscular reconditioning via supramaximal stimulation over a 6-week period (DiMarco et al., 2006).

Ultimately, however, combined dual-contact stimulation of the T9- and L1- spinal levels achieved near-optimal cough performance, effectively improving Paw to 132 cm H_2_O compared to 90 cm H_2_O and 82 cm H_2_O at each respective location individually. PEF was also augmented to 7.4 L/s compared to 6.4 L/s and 5.0 L/s at each respective location individually (DiMarco et al., 2006). Consistent with preclinical results, additional co-stimulation via a third lead at the T11-level did not result in further improvement. The researchers concluded that overall the pilot study supported the proposed two-electrode set-up targeting the T9- and L1- spinal levels as potentially adequate for generating clinically sufficient cough performance. Importantly, the subject gained ability to use the device independently for triggering cough thus alleviating caregiver-dependence (DiMarco et al., 2006).

This encouraging pilot study led to a clinical trial in nine individuals with cervical SCI and significantly impaired cough function, defined as reduced maximum expiratory pressures <30 cm H_2_O and PEF rates <2.5 L/s. The device was capable of triggering forceful cough in all subjects. The proposed dual-contact stimulation approach proved efficacious for achieving near-maximal cough performance with evoked Paws ranging from 162 cm to 206 cm H_2_O and PEFs ranging from 10.1 L/s to 10.6 L/s at total lung capacity (TLC), approximating performance levels of physiological cough in healthy volunteers (Leith et al., 1986). Generally, optimal expulsion of secretions or foreign bodies requires coordinated phasic activation of the inspiratory and then expiratory compartments following the phases of physiological cough cycle (Loudon and Shaw, 1967). Consequently, device users were trained to perform volitional maximal inspiration, briefly hold their glottis closed (i.e., hold their breath) before triggering the stimulating device while trying to forcefully exhale within 1 s following the stimulus (DiMarco et al., 2009a,b). Users can thus control the device independently for triggering cough.

Achieving maximal Paws also requires coordinated neuromuscular activation with maximal forceful contraction of all major expiratory muscles (Siebens et al., 1964; Langlands, 1967; Loudon and Shaw, 1967; Arora and Gal, 1981). Consequently, the robust performance on these key expiratory measures (i.e., Paw and PEF) in the SCI subjects suggests that dual stimulation successfully evoked total or near-total contraction of the major expiratory contributors. Conversely, single site stimulation resulted in markedly reduced performance with Paws ranging from 120 cm to 144 cm H_2_O and PEFs ranging from 5.8 L/s to 8.6 L/s, supporting the concept that dual-contact stimulation may be required for achieving adequately comprehensive expiratory recruitment at feasible and safe stimulus parameters. Interestingly however, in contrast to the preclinical studies, performance at each individual contact did not differ significantly from one another, resulting in overall similar magnitudes of airway pressure. Various combinations of any two contacts evoked equivalent cough performance in the human subjects. Finally, as in the preclinical and pilot studies, co-activation of all three contacts did not evoke further improvement of cough performance over two contacts (DiMarco et al., 2009a,b).

Additionally, airway pressures and peak airflow rates correlated linearly and similar to physiological cough performance markers of stimulation-evoked cough also correlated with baseline air volumes. Consequently, compared to TLC the performance measures from functional residual capacity were reduced with Paws of 107–134 cm H_2_O and PEFs of 6.7–7.7 L/s, albeit nonetheless still markedly elevated from baseline and adequate for expected clinical benefit. Of note, studies of patients with Duchenne muscular dystrophy have estimated that during upper respiratory infection, a minimum PEF of 4.5 L/s is required to prevent respiratory failure (Bach et al., 1997). Thus, the PEF achieved via lower thoracic SCS far exceeded the estimated minimum threshold levels even at lower baseline lung volumes.

Finally, evoked cough performance also correlated with applied stimulus parameters. Evoked Paws thus increased with higher stimulus amplitudes before eventually plateauing at approximately 30–40 V via the two-electrode set-up. Similarly, evoked Paws also increased with higher frequencies before reaching a plateau at approximately 40–50 Hz. Conversely, pulse widths above 150 μs did not result in further increase of airway pressures (DiMarco et al., 2009a,b). Compared to baseline cough insufficiency measures (i.e., <30 cm H_2_O/<2.5 L/s), stimulation-evoked performance of 162–206 cm H_2_O/10.1–10.6 L/s (at TLC) represents an increase of up to 5–7 fold for Paw and ~4-fold for PEF. Cough performance far exceeded the reported results of peripheral abdominal stimulation approaches that have shown only modest increases in Paw by ~50% (i.e., ~30 cm H_2_O) to maximal 55–60 cm H_2_O (Jaeger et al., 1993; Linder, 1993) and of peak flow-augmentation by up to ~80% (Butler et al., 2011).

Long-term follow-up of this clinical trial has since been reported and has shown that lower thoracic SCS remains safe and effective over the mean follow-up of 20.0 ± 3.7 months (DiMarco et al., 2009a,b). At 40 weeks it was found that need for suctioning and assisted cough to clear secretions was virtually abolished in device-users resulting in a significant decrease of caregiver assistance from an average of 16.9 ± 7.9 times/week to 0.4 ± 0.3 times/week (DiMarco et al., 2009a,b).

User experience with the device was assessed through rating scales and self-report. In general, subjects reported that their condition interfered less with daily activities and family life and that they experienced improved control over breathing and reduced stress and embarrassment from respiratory problems and the need for coughing assistance. Self-ratings of difficulty raising sputum and clearing secretions were consistently reported as having improved from “moderate” or “marked” to “mild” or “no difficulty.” Severity of coughing episodes was also reported as improved from “moderate” (i.e., interrupting activities) to “mild” or “none.” Finally, subjects also reported marked improvement in mobility. Because the device can be independently activated by the person as needed without caregiver assistance, it enabled more independence for activities like travel without caregivers (DiMarco et al., 2009a,b, 2014).

At maximal follow-up of 4.6 years, all study subjects continued regular use of the device, and the mean stimulation-evoked maximum airway pressure remained markedly elevated at 108 ± 23 cm H_2_O, stable and not significantly different from the 1-year mark of 103 ± 20 cm H_2_O. Moreover, benchmark improvements in quality of life and respiratory care remained elevated and the need for trained caregiver support and alternative secretion management were markedly reduced allowing caregiver-free travel in five of the nine subjects. Use of the device also significantly reduced the severity and incidence of respiratory tract infections from baseline 1.4 ± 0.3/subject year to 0.2 ± 0.1 (DiMarco et al., 2014).

### Mechanisms of Stimulation-Evoked Expiratory Motor Recruitment

Electrical current applied within the epidural space is known to propagate within the cerebrospinal fluid (CSF) space to depolarize adjacent nerve roots at a segmental level. The previously mentioned studies of lower thoracic SCS (DiMarco et al., 1999a,b, 2006, 2009a,b; DiMarco and Kowalski, 2008) consistently resulted in local motor root recruitment with evoked segmental short-latency compound action potentials (CAPs) of the surrounding nerve roots. These changes probably relate to immediate electrical field effects and direct depolarization in that sufficiently high current amplitudes could directly recruit nerve roots across multiple spinal levels in either direction via immediate current spread (e.g., across two to three spinal levels depending on stimulus intensity). Interestingly, the stimulus-response curve showed an initial sharp increase in evoked Paw from 0 mA to 15 mA before then flattening out at higher amplitudes (i.e., 15–50 mA; DiMarco et al., 2006, 2009a,b; DiMarco and Kowalski, 2008).

It is now generally accepted that epidural stimulation is also capable of modulating intrinsic spinal pathways and interneuronal connections exerting either facilitative or inhibitory effects on motor output. Given adequate stimulation, these circuits are thus potentially capable of mediating more widespread and remote network effects, possibly including those of functionally connected segments of the nervous system distant to the stimulus source. For example, DiMarco et al. (2002) found in their animal studies that stimulation of the T9/10-spinal level evoked not only local-segmental short-latency CAPs at the T9/10 motor roots, but also long-latency (i.e., >3.6 ms) CAPs at the T11–L2 motor roots consistent with additional indirect recruitment of remote-caudal motor roots. This indirect recruitment effect of the more caudal motor roots provided a substantial contribution to stimulation-evoked expiratory performance. Consistently, sectioning of the T8–T10 motor roots had profound impact on evoked expiratory pressures. However, abolishing the remote long-latency CAPs via either sectioning of the T11–L2 roots or sectioning of the dorsal columns, caused the greatest reduction of stimulation-evoked Paws (up to a 60%–80% decrease relative baseline; DiMarco et al., 2002). Additional sectioning of the lateral and ventral funiculi diminished the evocable Paws by additional 16% and 12%, respectively, implicating at least partially these fiber tracts (spinocerebellar, corticospinal, rubrospinal, and/or propriospinal tracts) in mediating the observed remote motor network effects.

Overall, these findings are consistent with studies from the application of epidural stimulation to respiration or limb motor function. Stimulation-evoked modulation of the afferent dorsal root fibers may be involved in mediating these functional network effects for remote motor recruitment, possibly via mono- or polysynaptic integration at the level of the dorsal column and/or via complex interneuronal networks potentially spanning across several spinal segments. In conclusion, these experiments by DiMarco et al. (1995, 1999a,b, 2002, 2006, 2009a,b, 2014) have demonstrated that epidural SCS of the lower thoracic spinal cord (e.g., T9–T13 spinal levels) is capable of generating comprehensive and near-complete recruitment of expiratory musculature with clinically significant pressure gradients for forceful expiratory air flow mimicking physiological cough.

### Limitations and Challenges

There remain some limitations and challenges to be met in epidural SCS for cough. Lower thoracic SCS requires relatively high amplitudes to achieve adequate motor recruitment and contraction force. High stimulus intensities can cause relatively broad disseminated current spread across multiple spinal levels, thereby evoking relatively nonspecific activation of adjacent nerve roots. Untargeted current spread may depolarize surrounding axons, adjacent nerve roots or spinal tracts, as well as recruit unrelated intraspinal neural circuits that can cause remote effects. Overall, these studies have suggested that stimulation above 15 mA increased the risk for adverse effects related to untargeted spillover current spread. For example, undesired muscle contractions/spasms of the paraspinal and/or thigh muscles resulting in some trunk motion and leg jerks have occurred in all subjects with stimulation at the L1-spinal level. Stimulation at the T11-segment caused only mild leg twitches, whereas stimulation at the T9-level was not associated with apparent muscle contractions. The resulting safety and feasibility limitations imposed on amplitude ranges thus prohibited a single-electrode paradigm capable of evoking the motor recruitment needed for sufficient contraction force. While the reported leg jerks were generally well tolerated and not associated with pain or discomfort, nonspecific stimulation remains an inherent limitation of epidural SCS (DiMarco et al., 1999a,b, 2006, 2009a,b; DiMarco and Kowalski, 2008).

During the initial conditioning phase over the first weeks, three out of nine subjects with cervical injury also experienced mild hemodynamic changes characteristic of autonomic dysreflexia, however these changes ceased without intervention within ~7 min of terminating stimulation. Moreover, the autonomic changes did not evoke any of the typical symptoms commonly associated with autonomic dysreflexia (e.g., headache, flushing and sweating). These effects subsequently diminished and ultimately subsided following daily stimulation over a period of several weeks. No bowel or bladder dysfunction occurred in any of the subjects (DiMarco et al., 2009a,b).

## High-Frequency Spinal Cord Stimulation (HF-SCS) for Cough Restoration

Lower thoracic SCS at 50 Hz has shown robust activation of expiratory muscles with efficacious airway pressures in ranges characteristic of clinically feasible cough, albeit at relatively high stimulus amplitudes. In the reviewed studies of lower thoracic SCS at 50 Hz, particularly with amplitudes above 15 mA/30–40 V, undesired motor effects (e.g., twitches, jerks and spasms), sensory effects (e.g., dysesthesias or nociception) and autonomic effects (e.g., autonomic dysreflexia) have occurred. Although well tolerated by the individuals and reversible with cessation of stimulation (DiMarco et al., 2009a,b), nonspecific adverse stimulation effects associated with high amplitudes remain a notable limitation of this approach and one that may ultimately limit provider and patient acceptance for widespread clinical implementation.

Efforts to restore inspiratory function using high-frequency SCS (HF-SCS) of the upper thoracic (T2) spinal cord at 300 Hz have shown promising preclinical results for respiratory restoration via intercostal pacing with potential phrenic co-activation (Decima et al., 1967, 1969; Decima and von Euler, 1969; DiMarco and Kowalski, 2009, 2010; DiMarco et al., 2009b). Based on these promising findings of upper thoracic HF-SCS, DiMarco and colleagues (Kowalski et al., 2016b) recently also started investigating the application of HF-SCS to the lower thoracic spinal cord for cough restoration. In the canine animal model, they found that stimulation at 500 Hz at the T9-spinal level could evoke robust expiratory performance (mean positive airway pressures of 58 ± 4 cm H_2_O) with amplitudes as low as 1 mA [64]. In contrast, 50 Hz stimulation required an amplitude of 15 mA to evoke equivalent airway pressures (~60 cm H_2_O). Stimulation above and below 500 Hz resulted in declining performance (Kowalski et al., 2016b). Presently, the mechanisms of activation and neural circuits involved in mediating these high frequency effects are unknown. Further investigation is warranted to ascertain the mechanisms, efficacy and safety of this promising new HF-SCS paradigm so as to expedite its translation into the clinical investigative setting and work toward developing an integrated dual-system approach for respiratory and cough restoration.

## Conclusions

The results of the preclinical studies and the clinical trial of nine subjects suggest that epidural SCS of the lower thoracic spinal cord holds promise for cough restoration in individuals with cervical SCI, evoking near-maximal expiratory cough performance with positive airway pressures and peak flow rates approximating physiological cough (DiMarco et al., 1995, 1999a,b, 2005, 2009b). Moreover, long-term follow-up of this initial clinical trial has demonstrated its sustained efficacy in alleviating respiratory complications, thus potentially reducing medical intervention and health care costs as well as the need for trained caregiver assistance. Subjects reported improved sense of well-being and quality of life, better control of breathing problems, increased ease and independence in removing secretions, reduced stress, and a greater sense of autonomy and mobility. The findings also supported the safety and efficacy of this new approach over 4.6 years of regular device usage (DiMarco et al., 2009b).

However, at intermediate frequencies of 50 Hz, lower thoracic SCS required relatively high amplitudes (30–40 V) via multiple electrodes to achieve the muscle recruitment and contraction force necessary for adequate cough performance. Unfortunately, amplitudes of that magnitude can potentially cause a number of adverse effects, including muscle spasms, dysesthesias, nociception, or dysreflexia, which may or may not be tolerated.

To overcome this important limitation, higher stimulation frequencies have been explored. Stimulation of the lower thoracic segments at 500 Hz has shown promise as a viable alternative in that the amplitude required for equivalent clinical outcomes was reduced 15-fold. The reduction in stimulation amplitude of frequencies at 500 Hz could potentially reduce or eliminate the adverse effects of spillover current spread, while reducing energy demands and lengthening battery life. Understanding the differences in spinal activation and neuromuscular recruitment patterns between stimulation at 50 Hz and at 500 Hz will be a critical step in establishing the long-term efficacy and safety of this new paradigm. Given that commercially available FDA-approved epidural stimulation devices, which are widely applied to alleviate chronic pain and spasticity, have already demonstrated long-term efficacy and safety of these devices (Turner et al., 2004; Taylor et al., 2006), it is possible that existing SCS devices could therefore be adapted into novel applications such as functional restoration of cough.

## Author Contributions

JTH was responsible for conceptualization and drafting. JTH, JSC, PJG, DID, KHL and IAL were responsible for editing, critical revision, final draft approval and submission. JTH and JSC created the figures.

## Conflict of Interest Statement

The authors declare that the research was conducted in the absence of any commercial or financial relationships that could be construed as a potential conflict of interest.
